# Temporal changes in cortical sensory processing during the transition from acute to chronic low back pain

**DOI:** 10.1097/PR9.0000000000001269

**Published:** 2025-04-24

**Authors:** Wei-Ju Chang, Luke C. Jenkins, Peter Humburg, Siobhan M. Schabrun

**Affiliations:** a Centre for Pain IMPACT, Neuroscience Research Australia (NeuRA), Randwick, New South Wales, Australia; b School of Health Sciences, Faculty of Health and Medicine, University of New South Wales, UNSW Sydney, New South Wales, Australia; c School of Health Sciences, Western Sydney University, Penrith, New South Wales, Australia; d Stats Central, Mark Wainwright Analytical Centre, University of New South Wales, UNSW Sydney, New South Wales, Australia; e School of Physical Therapy, University of Western Ontario, London, ON, Canada; f The Gray Centre for Mobility and Activity, Parkwood Institute, St. Josephs HealthCare, London, ON, Canada

**Keywords:** Low back pain, Sensory evoked potential, Electroencephalography, Somatosensory cortex, Quantitative sensory testing

## Abstract

**Introduction:**

Sensory cortex activity differs between acute and chronic low back pain (LBP) with decreased activity in acute LBP but increased activity in chronic LBP. However, it is unknown how and when sensory cortex activity changes during the transition from acute to chronic LBP and whether fluctuations in sensory cortex activity are associated with pain sensitivity.

**Objectives:**

To examine (1) the trajectory of changes in sensory cortex activity over a period of six months from the onset of acute LBP; (2) the assoication between sesnory cortex activity and pain pain sensitivity.

**Methods:**

In a prospective cohort study (N = 120), sensory evoked potentials (SEPs) were recorded using electroencephalography within 6 weeks of LBP onset (baseline) and at 3- and 6-month follow-up. Pain sensitivity was assessed using quantitative sensory testing (QST).

**Results:**

At baseline, individuals who developed chronic pain at 6-month follow-up exhibited less nonnoxious sensory processing (smaller area of N_80_, N_150_, and P_260_ SEP components) compared with those who recovered. In individuals who recovered, SEP measures did not change over time. In individuals who developed chronic pain, N_80_ and N_150_ SEP area increased between baseline and 3 months, while P_260_ SEP area increased between baseline and 6 months, reaching values consistent with those of recovered individuals. Sensory evoked potential measures showed no relationship with QST, except for a larger P_260_ SEP area correlating with higher 6-month heat pain threshold at the site of LBP.

**Conclusions:**

Nonnoxious sensory processing in acute LBP was less in those who developed chronic pain than those who recovered. Nonnoxious sensory processing increased over time in those who developed chronic pain but was unchanged in those who recovered. In the transition from acute to chronic LBP, nonnoxious sensory processing in the sensory cortex was not associated with pain sensitivity.

## Abstract 2

Supplemental Digital Content is Available in the Text.

In people with an acute episode of low back pain, how processing of nonnoxious sensory inputs changed over time differentiated their 6-month pain, but not disability outcome.

## 1. Introduction

Despite decades of research, mechanisms underlying symptom persistence in chronic low back pain (LBP) remain unclear. Emerging evidence suggests that altered cortical processing of sensory inputs is a mechanism linked to the development of chronic LBP.^17,34,51^ People with chronic LBP exhibit greater primary somatosensory cortex (S1) activity in response to noxious stimuli than pain-free controls, and stronger S1 activity is related to longer pain duration and more severe pain.^23,25^ Conversely, individuals who display less processing of nonnoxious sensory stimuli in the acute stage of LBP are more likely to develop chronic LBP than those who display higher sensory processing.^34,35^ However, how and when cortical processing of sensory inputs changes during the transition from acute to chronic LBP is unknown.

Cortical processing of sensory inputs has been examined using sensory evoked potentials (SEPs) recorded by electroencephalography (EEG).^23,50,51^ Neuroimaging evidence suggests that distinct SEP components correspond to sensory processing in specific cortical areas: the N_80_ from S1, N_150_ from secondary sensory cortex (S2), and P_260_ from anterior cingulate cortex (ACC).^9,10,59,60^ Thus, these SEP components have been used to index somatosensory and cingulate cortex activity.^14,20,23,34,35^ Functionally, S1 and S2 are involved in sensory discrimination,^52,66^ while S2 and ACC integrate nociceptive and nonnociceptive inputs with roles in the emotional and motivational aspects of pain perception.^3,26,60^ In chronic LBP, greater N_80_ (S1 activity) and smaller P_260_ (ACC activity) amplitudes evoked by painful stimuli were observed relative to pain-free controls, indicating increased sensory discrimination and decreased processing of the emotional and motivational dimension of pain respectively.^23^ In acute LBP, N_150_ and P_260_ amplitudes evoked by nonnoxious stimuli were smaller than pain-free controls, suggesting less processing of nonnoxious sensory inputs in S2 and ACC.^14^ Further, S2 and ACC activity in acute LBP was associated with symptoms: those with lower S2/ACC activity reported more severe pain than those with higher S2/ACC activity.^14^ How cortical processing of sensory inputs changes over time after the onset of acute LBP may differ between individuals, potentially influencing clinical outcome.

Preliminary evidence suggests an association between cortical sensory processing and pain hypersensitivity,^21^ a characteristic thought to indicate central sensitisation (defined as “an amplification of neural signalling within the central nervous system to elicit pain hypersensitivity”), a mechanism associated with chronic LBP.^64^ Indeed, research has shown greater cortical sensory processing in patients with chronic pain who also exhibited pain hypersensitivity when compared with controls.^27,29,41^ Further, greater S1 activation on functional magnetic resonance imaging correlates^27^ while a greater S1 activity is associated with electrical pain hypersensitivity in chronic LBP.^23^

We aimed to examine (1) the trajectory of changes in SEP components as people either transitioned to chronic LBP or recovered over a period of 6 months from the onset of acute nonspecific LBP; (2) the association between SEP and measures of central sensitisation (eg, pressure [PPTs] and heat pain thresholds [HPTs] and conditioned pain modulation [CPM]).

## 2. Methods

### 2.1. Participants

This study is a secondary analysis of the UPWaRD study—a prospective longitudinal cohort study (Trial Registration Number ACTRN12619000002189) investigating biopsychosocial risk factors of poor 6-month outcome in 120 people with acute nonspecific LBP.^33^ The UPWaRD study was conducted between December 2014 and July 2019 at Western Sydney University and Neuroscience Research Australia in Sydney, Australia. Participants were recruited through newspaper/on-line advertisements, flyers and social media sites, primary health care professionals, and local hospitals in Southeast and Southwest Sydney Local Health Districts. Ethical approval was obtained from the Western Sydney University (H10465) and Neuroscience Research Australia (SSA: 16/002).

Eligible participants were 18 years or older, currently experiencing acute nonspecific LBP, and could speak and read English adequately.^22^ Acute LBP was defined as pain located between the 12th ribs and gluteal fold, lasted for >24 hours and <6 weeks, preceded by a period of >1 month without LBP.^22,55,63^ Participants reported leg pain that was not radicular pain resulted from neural tissue involvement^62^ or lumbosacral radiculopathy^30^ remained eligible. If researchers, who were qualified physiotherapists, suspected lumbosacral nerve root involvement, they conducted detailed history-taking during eligibility screening and a physical examination at baseline assessment. Participants with radicular pain and/or lumbosacral radiculopathy were excluded. People with suspected serious spine pathology, other major diseases/disorders, a history of spine surgery, any other chronic pain conditions, or contraindications to the use of transcranial magnetic stimulation were excluded.^37^

### 2.2. Procedures

Participant's eligibility was determined by the researchers via telephone. Written informed consent was obtained from each participant upon their arrival at the baseline assessment. Participants were assessed at baseline, 3-, and 6-month follow-ups.

#### 2.2.1. Processing of nonnoxious afferent inputs

**Figure 1.**
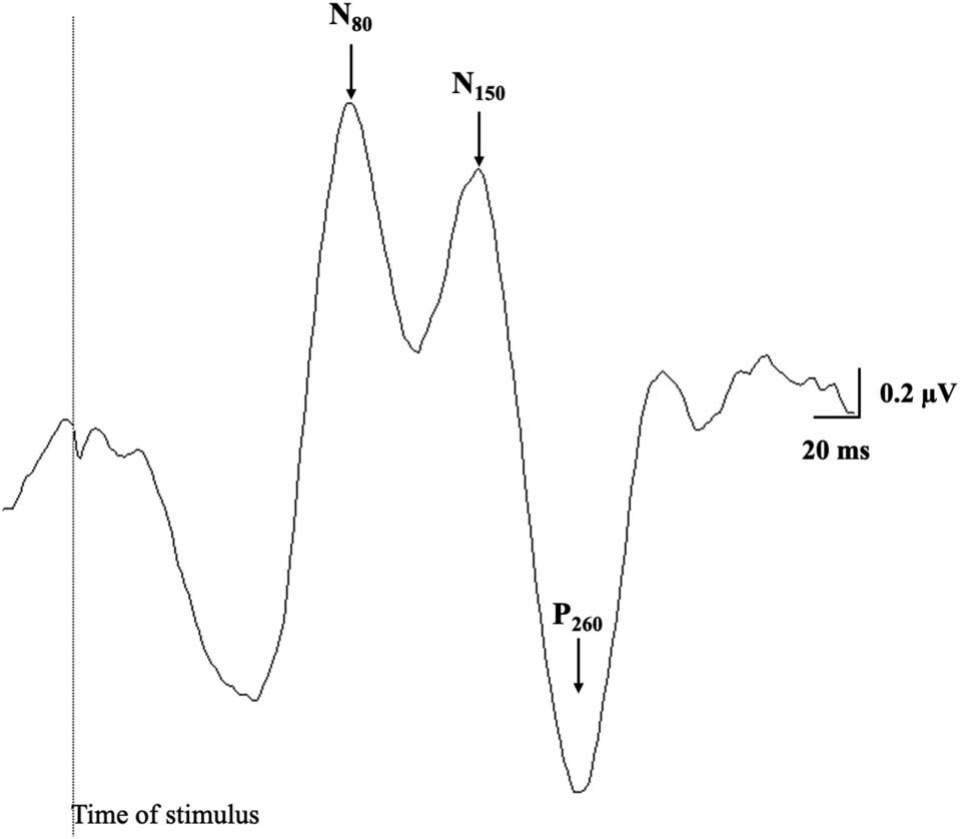
The raw data of a single participant to illustrate individual components of the sensory evoked potential.

Electroencephalography was recorded using gold plated cup electrodes over S1 (3 cm lateral and 2 cm posterior to Cz) on the side contralateral to the side of worst pain and referenced to Fz using the International 10/20 System.^51^ Previous research found that SEP was best detected at S1 contralateral to the electrical stimulation site.^23^ Electrode impedance was kept <5 kΩ. Electroencephalography signals were amplified 50,000x, filtered between 5 and 500 Hz, and sampled at 1000 Hz using Micro1401 data acquisition system and Signal software (CED Limited, Cambridge, United Kingdom). Participants were seated with eyes closed and instructed not to fall asleep during EEG recording while received 2 blocks of 500 nonnoxious electrical stimuli (pulse duration:1 ms, frequency: 2 Hz, maximum current: 1A; Digitimer, DS7AH) on the side of the worst LBP (3 cm lateral to the L3 spinous process). Stimulus intensity was set at 3x perceptual threshold and adjusted where necessary to ensure the stimuli were nonnoxious. A 20% variance was incorporated into the stimulus frequency to reduce accommodation. Sensory evoked potentials were analysed as area for N_80_ (between the first major downward deflection of the curve after stimulation and the first major negative peak, N_80_), N_150_ (between the first negative peak, N_80_ and second negative peak, N_150_), and P_260_ (between the second negative peak, N_150_ and the positive deflection of the curve starting around 150 ms after stimulus onset, P_260_) components (Fig. 1).^23,51^ Sensory evoked potential areas were averaged across the 2 SEP blocks.

#### 2.2.2. Quantitative sensory testing

Heat pain threshold and pressure pain threshold were assessed at the site of worst LBP to index localised pain sensitivity and at forearm/thumb to index widespread pain sensitivity.^15^ Conditioned pain modulation was used to assess descending pain modulation that facilitates or inhibits pain.^38,65^ A deficient CPM response indicates impaired descending pain inhibitory control.^38^

*Heat pain thresholds* were assessed using the Thermal Sensory Analyzer (TSA-2001, Q-Sense-CPM, Medoc Ltd, Ramat Yishai, Israel) via a 30 × 30 mm thermode applied to the skin's surface and the temperature increased from 32°C at a rate of 1°C/second. Participants lie prone with arms by their sides and pressed a button when the sensation of heat first became the sensation of pain. Threshold values were truncated if participants did not report a sensation of pain by 50°C. Heat pain thresholds were recorded at (1) 3 cm lateral from the L3 spinous process on the side of the worst pain, (2) 3 cm lateral from the L3 spinous process on the side opposite to the worst pain, and (3) the ventral aspect of the forearm contralateral to the side of the worst pain, with a 30 seconds rest period between each trial. Three measures at each site was averaged for analysis. Averaged HPTs at sites (2) and (3) were also used in the subsequent CPM assessment. Heat pain thresholds have shown excellent relative reliability (intraclass correlation coefficient [ICC] = 0.87) in LBP population.^48^

*Pressure pain thresholds* were assessed at 2 sites: (1) the site of worst LBP and (2) the thumbnail ipsilateral to the worst site of LBP, with a 30-second rest between assessments by a hand-held pressure algometer with a 1 cm^2^ surface probe (Somedic, Hörby, Sweden) to apply pressure perpendicular to the skin's surface at a rate of 40 kPa/second. Participants press a button when the sensation of pressure first became a sensation of pain. Three recordings at each site were averaged for analysis. Pressure pain thresholds have shown excellent relative reliability (ICC = 0.97–0.99) in LBP population.^6,48^

*Conditioned pain modulation* was quantified as a change in PPT (test stimulus [TS]) at 1 body site in the presence of pain induced by a noxious heat stimulus (1°C above HPT for 30 seconds; conditioning stimulus [CS]) at another body site using the Thermal Sensory Analyzer.^65^ Three PPTs were measured before applying the heat stimulus (TS_1_). Heat increased from 36°C, at a rate of 0.5°C/second until HPT +1°C. Participants rated their pain on a numerical rating scale (NRS; 0 = “no pain,” 100 = “worst pain imaginable”) 30 seconds after the heat reached the plateau. Three PPTs were reassessed when heat pain scores were between 50 and 80 (TS_2_). The temperature was increased if heat pain scores were <50 or decreased if heat pain scores were >80, and the trial restarted. Participants completed 2 CPM trials in random order with a 15-minute wash-out period between-trials: (1) TS at the site of worst LBP (3 cm lateral from the spinous process of L3 vertebra) and CS on the contralateral forearm; (2) TS at the ipsilateral forearm (10 cm distal from the lateral epicondyle) and CS on the back contralateral to the side of the worst pain. The magnitude of CPM was determined as TS_2_ minus TS_1_, where a positive value indicated normal endogenous pain inhibitory function.^8^ The CPM paradigm has good relative reliability (ICC > 0.6).^42^

#### 2.2.3. Questionnaires

Demographics including age, sex, body mass index, socioeconomic status, and cultural and linguistic diversity were collected. Each participant's postal code was converted into a socioeconomic index for areas score, with higher scores being considered higher socioeconomic status for people living within that postal code.^5^ Participants with a cultural or ethnic background other than “English,” “Caucasian,” or “Australian” were considered culturally diverse. Participants reported any previous history of LBP, and use of health care and medication for their current LBP episode. Participants rated their pain on average over the previous week using an 11-point NRS (0 = “no pain,” 10 = “worst pain imaginable”). *The Roland Morris Disability Questionnaire* (*RMDQ,* 24 questions) was administered to assess the level of LBP-related disability (“0” = no disability, “24” = severe disability).^49^ *The Depression Anxiety Stress Scale-21* (*DASS 21*, 21 items) was used to measure negative emotional states of depression, anxiety, and stress.^47^ Higher subscale scores indicate more severe depression, anxiety, and stress.^43^ *The Pain Catastrophising Scale* (13 items) was used to assess participants' thoughts and feelings about pain in the domains of magnification, rumination, and helplessness.^46^ Higher scores indicate more severe pain-associated catastrophising. *The Pain Self Efficacy Questionnaire* (10 items, 0–6 points/item) was administered to assess a participant's confidence performing activities while in pain.^44^ Scores range from 0 to 60, with a cut-off value <40 representing low pain self-efficacy.^58^

### 2.3. Primary outcomes

The primary outcomes were pain intensity and disability at 6-month follow-up.^32^ Participants who reported a NRS score ≥1 at 6 months were considered to have developed chronic pain and those who reported a RMDQ score ≥3 at 6 months were considered to have developed chronic disability.^36^ These cut-off values were found to reflect patient expectations of complete recovery from a LBP episode.^36^

### 2.4. Statistical analysis

All statistical analyses were conducted using R, version 4.03 (R Development Core Team, Vienna, Austria).^18^ Statistical significance was set at *P <* 0.05. Descriptive analyses were used to report baseline demographics and participant characteristics. Mean and standard deviation (SD) for continuous variables and frequency (percentage) for categorical variables were presented. Baseline data were compared between those developed chronic pain or chronic disability and those recovered at 6 months using chi-squared (categorical variables) or independent t tests (continuous variables). Homogeneity was assessed using Levene test. Welch *t* test was performed if variables did not meet the equal variance assumption. As data were missing at random, missing data were imputed using the multiple imputation by chained equations procedure (R package MICE).^11^ Twenty imputed datasets were generated for analyses, and the results were pooled across analyses in each imputed dataset.

Linear mixed-effects models were used to examine changes in N_80_, N_150_, and P_260_ SEP areas over 3 time points (*time*: baseline vs 3-month vs 6-month) between LBP recovery status based on 6-month pain (*group*: recovered vs chronic pain) and disability (*group*: recovered vs chronic disability) (R package *lme4*).^7^ Separate models were fitted for individual SEP components. Independent variables included group, time, and group × time interaction term as fixed effects and participant-specific random intercepts. Residual plots were visually inspected for any obvious deviations from homoscedasticity. As log-transformation improved homoscedasticity, analyses were performed with N_80_, N_150_, and P_260_ SEP area log-transformed. To determine whether group × time interactions were significant, the models with and without the group × time interaction term were compared. Between-model differences (*P* < 0.05) indicate significant group × time interactions. Similarly, the significance of group or time main effects was assessed by comparing the models including the group × time interaction term vs the models excluding group or time accordingly. Further, pairwise comparisons between groups and time points were conducted. *P* values were corrected for multiple testing using Sidak adjustment. In addition, how and when pain and disability changed over time (*time*: baseline vs 3-month vs 6-month) were compared between those who recovered and those who developed chronic pain (*group*: recovered vs chronic pain) or chronic disability (*group*: recovered vs chronic disability) using this approach, with the models controlled for age as older age was a risk factor for poor outcome.^35^

### 2.5. Sensitivity analysis

To explore the influence of psychological factors on how cortical sensory processing, pain, and disability changed over 3 time points, we repeated the abovementioned analyses while controlling for DASS-21, pain catastrophizing scale (PCS), and pain self-efficacy questionnaire (PSEQ) scores.

To examine whether processing of nonnoxious sensory inputs was associated with quantitative sensory testing (QST) measures, separate linear mixed-effects models were fitted for log-transformed N_80_, N_150_, and P_260_ SEP area. Correlation coefficients between each QST measure at each time point were examined. All HPT (*r* = 0.54–0.69) and PPT (*r* = 0.52–0.70) measures had medium to strong correlations at each time point, whereas 2 CPM measures had very weak correlations (*r* = 0.05–0.16) (Supplementary File, http://links.lww.com/PR9/A302). Thus, we included HPT and PPT at the site of worst pain and 2 CPM measures as fixed effects and participant-specific random intercepts in the models.

## 3. Results

**Figure 2.**
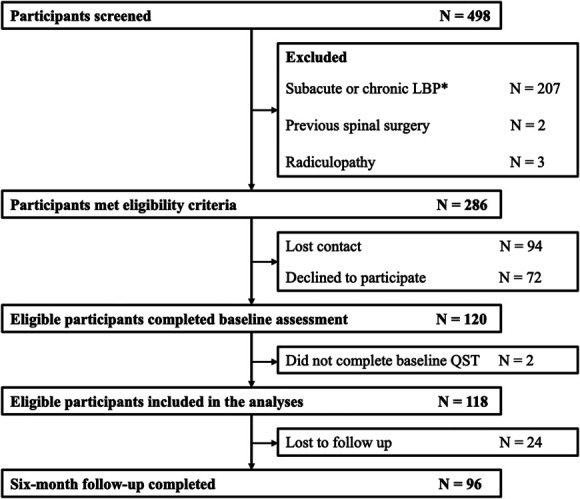
Study flowchart. *Defined as low back pain (LBP) that lasted for longer than 6 weeks and/or LBP episode preceded by pain-free for a period of less than 1 month.

The study flow is presented in Figure 2. Participant baseline characteristics are presented in Tables 1 and 2. Sensory evoked potential data were available from 118 participants (98.3%) at baseline, 74 (61.7%) at 3 months, and 72 (60%) at 6 months. Questionnaires were completed in all participants at baseline, 95 (79.2%) at 3 months, and 96 (80%) at 6 months. Regardless of whether 6-month outcome was defined by pain or disability, recovered participants had lower DASS-21 and PCS scores and higher PSEQ scores at baseline than unrecovered participants (*P* < 0.01).

**Table 1:** Baseline participant characteristics when outcome was defined by 6-month pain intensity.

	Recovered (N = 33)	Chronic pain (N = 87)	*P*
Sex: Female (%)	46.9	50.0	0.92
Age (y)*	34.3 (11.9)	41.7 (15.4)	**0.02**
Previous history of LBP: No (%)	25.0	19.3	0.92
Cultural diversity: No (%)	50.0	52.3	0.92
Socioeconomic status (SEIFA score)	1023.5 (51.1)	1025.3 (61.2)	0.92
PCS score*	6.2 (6.9)	12.7 (10.4)	**<0.01**
DASS-21 score*	10.4 (11.4)	24.3 (21.2)	**<0.01**
PSEQ score*	52.8 (8.2)	43.8 (12.3)	**<0.01**

Chronic pain was defined by the presence of pain (NRS ≥ 1) and recovery by the absence of pain (NRS = 0) at 6 months. Continuous data were presented as mean and standard deviation.

* Welch *t* test was performed. Bold fonts indicate *P* < 0.05.

DASS, depression, anxiety, stress scale; LBP, low back pain; PCS, pain catastrophizing scale; PSEQ, pain self-efficacy questionnaire; SEIFA, socioeconomic index for areas.

**Table 2:** Baseline participant characteristics when outcome was defined by 6-month disability.

	Recovered (N = 73)	Chronic disability (N = 47)	*P*
Sex: Female (%)	52.1	44.9	0.62
Age (y)*	36.8 (13.4)	43.9 (16.0)	**0.04**
Previous history of LBP: No (%)	23.9	16.3	0.38
Cultural diversity: No (%)	49.3	55.1	0.57
Socioeconomic status (SEIFA score)	1021.3 (58.4)	1030.1 (58.8)	0.54
PCS score*	8.3 (8.5)	14.9 (10.7)	**<0.01**
DASS-21 score*	14.1 (16.1)	30.1 (21.5)	**<0.01**
PSEQ score*	49.3 (10.6)	41.7 (12.5)	**<0.01**

Chronic disability was defined by the presence of disability (RMDQ ≥ 3) and recovery by the absence of disability (RMDQ ≤ 2) at 6 months. Continuous data were presented as mean and standard deviation.

* Welch *t* test was performed. Bold fonts indicate *P* < 0.05.

DASS, depression, anxiety, stress scale; LBP, low back pain; PCS, pain catastrophizing scale; PSEQ, pain self-efficacy questionnaire; SEIFA, socioeconomic index for areas.

### 3.1. Temporal profile of sensory evoked potential measures in the transition from acute to chronic low back pain

**Figure 3.**
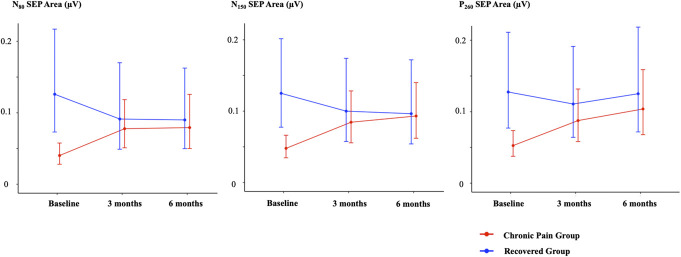
Changes in area of N_80_, N_150_, and P_260_ SEP components (mean and 95% confidence interval) over 6 months after an episode of acute low back pain when outcome was defined by chronic pain (NRS score ≥1 at 6 months). NRS, numeric rating scale; SEP, sensory evoked potential.

Sensory evoked potential areas are presented in Tables 3 and 4. There were no group × time interactions for the N_80_, N_150_, and P_260_ SEP areas, irrespective of whether outcome was determined by pain or disability (Supplementary File, http://links.lww.com/PR9/A302). When outcome was defined by pain, pairwise comparisons showed N_80_, N_150_, and P_260_ SEP areas were smaller at baseline in those who developed chronic pain at 6 months than recovered participants (adjusted *P* < 0.01) but did not differ at 3 or 6 months (adjusted *P* > 0.48). In those who developed chronic pain, N_80_ and N_150_ SEP areas increased between baseline and 3 months (adjusted *P* < 0.03) and were unchanged at 6 months (adjusted *P* > 0.92), while the P_260_ SEP area increased between baseline and 6 months (adjusted *P* = 0.01) (Fig. 3). When outcome was defined by disability, N_80_, N_150_, and P_260_ SEP areas did not differ between recovered and unrecovered participants at any time point (adjusted *P* > 0.19), and SEP areas did not change over time (adjusted *P* > 0.16).

**Table 3:** Group data (mean and standard deviation) for the measures of sensory evoked potential area and quantitative sensory testing when outcome is defined by pain at 6 months.

	Recovered (N = 33)			Chronic pain (N = 87)		
	Baseline	3 mo	6 mo	Baseline	3 mo	6 mo
N_80_ SEP area (µV)	0.37 ± 0.34	0.31 ± 0.51	0.26 ± 0.39	0.14 ± 0.27	0.24 ± 0.46	0.28 ± 0.54
N_150_ SEP area (µV)	0.32 ± 0.28	0.21 ± 0.30	0.20 ± 0.27	0.15 ± 0.27	0.22 ± 0.44	0.25 ± 0.49
P_260_ SEP area (µV)	0.35 ± 0.34	0.27 ± 0.47	0.29 ± 0.42	0.15 ± 0.27	0.22 ± 0.35	0.27 ± 0.45
HPT at the site of LBP (°C)	44.9 ± 2.7	45.0 ± 2.9	44.5 ± 2.0	45.3 ± 2.5	44.4 ± 2.7	44.7 ± 2.4
HPT at the contralateral lumbar site (°C)	45.1 ± 3.0	45.4 ± 2.4	44.2 ± 2.2	44.5 ± 2.9	44.1 ± 2.5	44.4 ± 2.4
HPT at forearm (°C)	44.1 ± 2.2	44.7 ± 1.9	44.7 ± 2.0	44.8 ± 2.2	44.2 ± 2.6	44.3 ± 2.5
PPT at the site of LBP (kPa)	943.0 ± 367.1	1103.1 ± 414.4	977.3 ± 299.6	835.0 ± 361.3	950.7 ± 472.2	905.9 ± 348.6
PPT at the thumb (kPa)	641.3 ± 269.0	679.0 ± 410.7	577.5 ± 190.3	638.5 ± 216.6	685.9 ± 308.2	651.7 ± 232.3
CPM—TS at the back (kPa)	20.9 ± 147.3	41.7 ± 198.8	39.4 ± 103.3	54.8 ± 160.5	67.4 ± 149.1	85.4 ± 158.5
CPM—TS at the forearm (kPa)	77.8 ± 151.0	92.7 ± 132.8	43.7 ± 114.0	39.5 ± 116.2	61.2 ± 136.9	46.6 ± 165.7

Chronic pain (N = 87) was defined by the presence of pain (NRS ≥ 1) and recovery (N = 33) by the absence of pain (NRS = 0) at 6-month follow-up.

CPM, conditioned pain modulation; HPT, heat pain threshold; LBP, low back pain; NRS, numeric rating scale; PPT, pressure pain threshold; SEP, sensory evoked potential; TS, test stimulus.

**Table 4:** Group data (mean and standard deviation) for the measures of sensory evoked potential area and quantitative sensory testing when outcome is defined by disability at 6 months.

	Recovered (N = 73)			Chronic disability (N = 47)		
	Baseline	3 mo	6 mo	Baseline	3 mo	6 mo
N_80_ SEP area (µV)	0.24 ± 0.31	0.32 ± 0.55	0.30 ± 0.53	0.15 ± 0.31	0.16 ± 0.27	0.24 ± 0.41
N_150_ SEP area (µV)	0.22 ± 0.27	0.26 ± 0.47	0.26 ± 0.50	0.17 ± 0.30	0.13 ± 0.18	0.18 ± 0.26
P_260_ SEP area (µV)	0.23 ± 0.30	0.28 ± 0.46	0.31 ± 0.46	0.18 ± 0.31	0.15 ± 0.22	0.22 ± 0.40
HPT at the site of LBP (°C)	45.3 ± 2.7	44.8 ± 2.9	44.7 ± 2.3	45.0 ± 2.4	44.3 ± 2.6	44.6 ± 2.3
HPT at the contralateral lumbar site (°C)	44.8 ± 3.1	44.8 ± 2.5	44.3 ± 2.4	44.5 ± 2.8	43.9 ± 2.4	44.4 ± 2.3
HPT at forearm (°C)	44.3 ± 2.2	44.4 ± 2.4	44.4 ± 2.4	45.0 ± 2.2	44.3 ± 2.5	44.4 ± 2.4
PPT at the site of LBP (kPa)	854.9 ± 352.5	1017.8 ± 418.7	941.7 ± 294.2	887.8 ± 388.2	964.1 ± 525.8	910.1 ± 388.8
PPT at the thumb (kPa)	641.4 ± 254.2	698.8 ± 365.7	629.8 ± 193.9	635.9 ± 193.4	657.1 ± 300.9	624.2 ± 260.0
CPM—TS at the back (kPa)	55.0 ± 135.9	76.4 ± 178.6	72.7 ± 140.0	27.8 ± 187.4	28.0 ± 132.9	68.1 ± 153.0
CPM—TS at the forearm (kPa)	61.5 ± 131.9	75.8 ± 143.8	28.3 ± 170.4	31.4 ± 118.1	60.2 ± 121.3	72.6 ± 112.0

Chronic disability (N = 47) was defined by RMDQ score of ≥3 and recovery (N = 73) by RMDQ score of ≤2 at 6-month follow up.

CPM, conditioned pain modulation; HPT, heat pain threshold; LBP, low back pain; PPT, pressure pain threshold; RMDQ, Ronald-Morrison Disability Questionnaire; SEP, sensory evoked potential; TS, test stimulus.

### 3.2. Temporal profile of pain and disability

**Figure 4.**
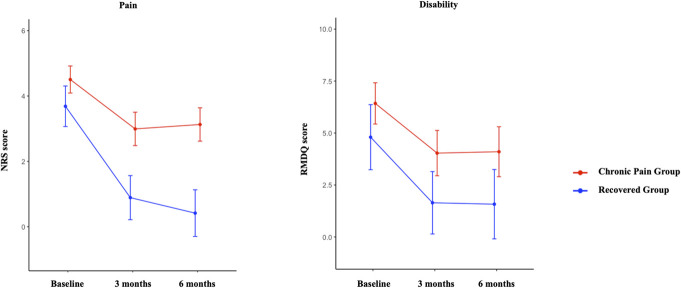
Changes in pain and disability scores (mean and 95% confidence interval) over 6 months after an episode of acute low back pain when outcome was defined by chronic pain (NRS score ≥1 at 6 months). NRS, numeric rating scale; RMDQ, Roland Morris Disability Questionnaire.

**Figure 5.**
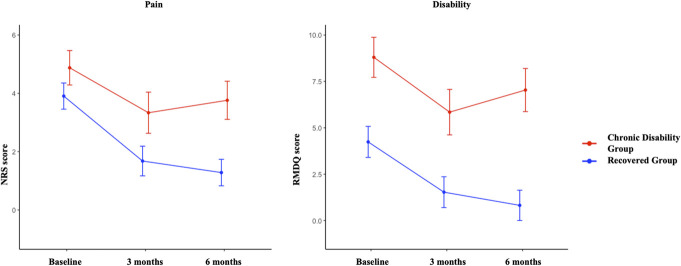
Changes in pain and disability scores (mean and 95% confidence interval) over 6 months after an episode of acute low back pain when outcome was defined by chronic disability (RMDQ score ≥3 at 6 months). NRS, numeric rating scale; RMDQ, Roland Morris Disability Questionnaire.

When 6-month outcome was defined by pain, there was a group × time interaction for pain but not for disability (Supplementary File, http://links.lww.com/PR9/A302). There were main effects of group and time for pain and disability. Pain and disability were higher in those who developed chronic pain than recovered participants at all time points (adjusted *P* < 0.03), except for that disability did not differ at baseline (adjusted *P* = 0.11) (Fig. 4). Pain (adjusted *P* < 0.01) and disability (adjusted *P* < 0.01) decreased between baseline and 3 months and remained unchanged between 3 and 6 months in both groups (adjusted *P* > 0.45). When 6-month outcome was defined by disability, there was a group × time interaction for pain but not for disability. There were main effects of group and time for pain and disability. Pain and disability were consistently higher in those who developed chronic disability than recovered participants at all time points (adjusted *P* < 0.03) (Fig. 5). Pain (adjusted *P* < 0.01) and disability (adjusted *P* < 0.01) decreased between baseline and 3 months and were unchanged between 3 and 6 months in both groups (adjusted *P* > 0.28).

### 3.3. Association between sensory evoked potential and quantitative sensory testing measures

The only SEP measure to show a relationship to pain sensitivity was P_260_ SEP area where a greater area was associated with a higher HPT at the site of worst pain at 6-month follow-up (*r* = 0.18, *P* = 0.04) (Supplementary File, http://links.lww.com/PR9/A302).

### 3.4. Sensitivity analysis

For SEP measures, controlling for psychological factors did not change the temporal profile when outcome was defined by pain. When outcome was defined by disability, controlling psychological factors showed smaller P_260_ SEP area at baseline in those who developed chronic disability than recovered participants. N_80_ SEP area increased between baseline and 6 months (adjusted *P* = 0.04) and P_260_ SEP area increased between baseline and 3 months (adjusted *P* = 0.02) and remained unchanged at 6 months (adjusted *P* = 0.81) in those developed chronic disability. For pain and disability, controlling psychological factors did not influence the temporal profile regardless of how 6-month outcome was defined.

## 4. Discussion

Our findings indicate cortical processing of nonnoxious sensory inputs might relate to LBP outcome. First, acute-stage N_80_, N_150_, and P_260_ SEP areas were smaller, reflecting reduced cortical sensory processing, in people who developed chronic LBP at 6-month follow-up compared with those who recovered. A new finding was that N_80_, N_150_, and P_260_ SEP areas increased over time in people who developed chronic pain, reaching values consistent with those of recovered individuals at 6-month follow-up, suggesting a normalisation of cortical sensory processing over time despite the presence of LBP. With the exception of a relationship between larger P_260_ SEP area and lower localised heat pain sensitivity at 6 months, cortical sensory processing was unrelated to QST measures.

In acute LBP (“baseline”), N_80_, N_150_, and P_260_ SEP areas were smaller in people who developed chronic pain than those who recovered, suggesting that lower sensory and cingulate cortex activity in acute LBP may contribute to pain chronicity. In contrast, SEP areas at baseline did not differ based on 6-month disability outcome. The discordance between pain- and disability-related findings is common in the literature, likely attributed to the low to moderate correlation between pain and disability observed in chronic LBP, suggesting different mechanisms may underpin these constructs.^40^ It is plausible that sensory cortical processing has stronger effects on pain perception than disability, which is heavily influenced by psychological factors such as pain-related psychological distress and negative pain beliefs.^1,12^

Previous neuroimaging research showed decreased S1, S2, and ACC activity in response to acute experimental pain, indicating the involvement of these cortical regions in the processing of acute pain.^3^ Acute pain could attenuate cortical activity via the “competing demands of pain” theory in which pain is thought to interrupt cognition by directing attention to noxious sensory input^13,53^ and competing with the processing of nonnoxious sensory inputs by shifting attentional resources.^4,24^ Indeed, a previous cross-sectional study showed that people with acute LBP had smaller N_150_ and P_260_ SEP areas (evoked by nonnoxious stimuli) compared with controls, suggesting acute LBP “distracts” the brain from the processing of nonnoxious sensory inputs.^14^ Alternatively, acute LBP could attenuate the processing of nonnoxious sensory inputs, akin to a “touch gating phenomenon,” whereby the presence of painful stimuli (acute LBP) weakens tactile stimuli (nonnoxious electrical stimulation), independent of an individuals' cognition.^2,14,28^ Importantly, our recent causal mediation analysis of the UPWaRD data found low acute-stage S1/S2 activity increased the odds of developing chronic LBP at 6 months, suggesting greater diminution in processing of nonnoxious sensory inputs in S1/S2 during acute LBP may be a causal mechanism of chronic LBP.^34^

Despite previous cross-sectional evidence in acute LBP that people with low S2/ACC activity experienced more pain in the past week than those with high activity, supporting the hypothesis of competing demands of pain,^14^ our data indicate no association between pain intensity and cortical sensory processing. Although acute-stage N_80_, N_150_, and P_260_ SEP areas differed between people who developed chronic pain and those who recovered, acute pain intensity did not differ. Further, despite differences in 3- and 6-month pain between those who developed chronic pain and recovered participants, SEP areas did not differ. Previous research suggests that an individual's pain rating is shaped by both sensory processing and pain expectation, underpinned by different brain mechanisms.^45^ It is plausible that SEP measures might capture mechanisms underlying sensory processing but not mechanisms underpinning an individuals' pain expectation (eg, cognitive evaluation, pain affect, and rewarding) as they occur in deeper brain regions and are not easily recorded on EEG.^31,54^

Our data reveal an overall increase in N_80_, N_150_, and P_260_ SEP areas over time after the onset of acute LBP in people who developed chronic pain but no change in those who recovered. While acute-phase SEP areas in those who developed chronic pain were smaller than those who recovered, the between-group differences diminished at 3 months. Further, 3-month SEP areas in those who developed chronic pain and baseline SEP areas in those who recovered did not differ, suggesting a normalisation of sensory processing by 3-month follow-up. Further, those who developed chronic pain consistently reported higher pain intensity over 6 months. Despite an increase over time in cortical processing of nonnoxious inputs, pain reduced but did not resolve in those who developed chronic pain. It appears that “normalisation” of cortical sensory processing might be insufficient to diminish pain, and other mechanisms (eg, psychological factors) might have a stronger influence on pain intensity.^19^ Although low acute-phase S1/S2 activity is causally related to chronic LBP, representing a novel treatment target,^34^ our findings suggest that early interventions targeting psychological factors might have stronger effects on prognosis than targeting somatosensory cortical activity.

Ongoing nociceptive input could increase the responsiveness of nociceptive neurons in the central nervous system to sensory inputs (termed “central sensitisation”),^61^ manifested as heightened pain responses and hypersensitivity to noxious and innocuous stimuli. Neuroimaging data suggest the association of central sensitisation with altered cortical sensory processing.^29^ Our analysis revealed that greater processing of nonnoxious inputs in ACC (larger P_260_ SEP area) was related to lower heat pain sensitivity (higher HPT) at the site of LBP at 6 months. This finding suggests the association between less pain-related emotional regulation and higher localised pain sensitivity at 6 months as ACC is involved in the affective aspect of pain perception and the assessment and regulation of emotion,^26,56,57^ but the contribution to long-term LBP outcome cannot be ascertained. While pain hypersensitivity was presented in subgroups of acute LBP,^16,39^ it was unrelated to acute-LBP cortical sensory processing in this study. Overall, we found no association between cortical sensory processing and pain sensitivity during the development of chronic LBP.

Our study have some limitations. First, missing data may introduce the attrition bias and might affect the robustness of our findings although they are inevitable in longitudinal studies. Multiple imputation was applied to reduce the impacts of removing participants with incomplete data from analyses. Second, we did not directly measure pain dimensions thought to be reflected on SEP components. Third, cortical sensory processing was measured only with nonnoxious stimuli. Future studies could assess the processing of noxious sensory inputs and multiple pain dimensions concurrently to elucidate how cortical sensory processing contributes to the development of chronic LBP. Finally, the relatively long experimental sessions (2–2.5 hours) might influence SEP and QST measures.

## 5. Conclusion

This study suggests that the temporal profile of sensory discrimination, the emotional and motivational dimensions of pain, and competition for cortical sensory processing might differ between individuals with acute LBP based on future pain outcome.

## Disclosures

The authors declare no financial arrangements that may represent a possible conflict of interest.

## Appendix A. Supplemental digital content

Supplemental digital content associated with this article can be found online at http://links.lww.com/PR9/A302.

## Supplementary Material

SUPPLEMENTARY MATERIAL
